# NCOR1 and NCOR2 Exhibit Distinct Cellular and Transcriptomic Signatures in Human Abdominal Aortic Aneurysm

**DOI:** 10.3390/biomedicines14040914

**Published:** 2026-04-16

**Authors:** Jaroslav Pelisek, Yankey Yundung, Anna-Leonie Menges, Fabian Rössler, Benedikt Reutersberg, Alexander Zimmermann, Martin Geiger

**Affiliations:** 1 Department of Vascular Surgery, University Hospital Zurich, 8091 Zurich, Switzerland; 2 Department of Surgery and Transplantation, University Hospital Zurich, 8091 Zurich, Switzerland

**Keywords:** nuclear receptor corepressors NCOR1 and NCOR2, abdominal aortic aneurysm (AAA), vascular cells, epigenetics, lncRNAs

## Abstract

**Background/Objectives**: Nuclear receptor corepressors NCOR1 and NCOR2 are key regulators of transcriptional repression, chromatin remodelling, and immunometabolic signalling. While NCOR1 has already been linked to vascular biology, its relevance in abdominal aortic aneurysm (AAA) remains unclear, particularly for NCOR2. This study aimed to investigate the expression, cellular localisation, and molecular interactions of NCOR1/2 in human AAA tissue. **Methods**: Human AAA samples (elective and ruptured) (*n* = 45) and non-aneurysmal control aortas (*n* = 18) were obtained from our Swiss Vascular Biobank. Transcriptomic profiling was performed using ribosomal RNA-depleted RNA sequencing. Differential expression and correlation analyses were performed using DESeq2/EdgeR and Spearman rank correlation with Benjamini–Hochberg correction. Cellular localisation was assessed through immunohistochemistry (IHC). **Results**: Bulk transcriptomic analyses showed no significant differences in NCOR1 or NCOR2 expression between AAA and controls. IHC revealed that NCOR1 was found in endothelial cells (ECs), smooth muscle cells (SMCs), and inflammatory infiltrates, while NCOR2 was primarily associated with macrophages. Correlation analyses suggest that NCOR1 interacts with various cellular markers, proteolytic enzymes, inflammatory mediators, and epigenetic regulators, including the lncRNA MALAT1. NCOR2 showed distinct associations with remodelling enzymes, TGFB1 signalling, selective epigenetic modifiers, and lncRNA H19. **Conclusions**: The lack of transcriptional differences in NCOR1 and NCOR2 between AAA and controls does not exclude cell-type-specific regulation or functional relevance. The specific cellular distributions and molecular associations in human AAA imply that NCOR1 and NCOR2 play non-redundant roles in vascular remodelling, inflammation, and epigenetic regulation. Our findings highlight NCOR pathways as potential modulators of AAA pathophysiology and promising targets for future therapies.

## 1. Introduction

Nuclear receptor corepressors NCOR1 and NCOR2, also known as N-CoR (nuclear receptor corepressor 1) and SMRT (silencing mediator of retinoid and thyroid receptors), are essential transcriptional coregulators of gene expression [1,2,3,4,5,6]. These proteins act as scaffolds for multi-protein corepressor complexes [1,5,7], which induce local chromatin modifications [1,3]. NCOR1/2 are also recognised as key factors in immunometabolic gene regulation, involving nuclear receptor signalling pathways that detect metabolic changes and regulate inflammation [1,6,8]. Furthermore, NCOR1 appears protective against atherosclerosis [9,10], whereas almost nothing is known about the role of NCOR2. Early mechanistic studies identified NCOR2 as a regulator of transcriptional repression of the inflammatory response [11]. Moreover, NCOR2 works alongside NCOR1 to control stimulus-dependent inflammatory signalling [12]. Current research describes NCORs as immunomodulators that coordinate inflammatory signalling, a process that plays an important role in many vascular pathologies.

Although *NCOR1* and *NCOR2* share similar functional domains, emerging evidence indicates that they play distinct roles and harbour distinct splice variants [13]. Furthermore, cell-type specificity has been observed [1,5]. Additionally, a study on endothelial cells found that NCOR1 plays a crucial role in angiogenesis, whereas NCOR2 had no effect [14]. Thus, NCOR1 and NCOR2 may have different target preferences depending on the cell type and signalling pathways.

As co-repressors, NCOR1 and NCOR2 are also essential epigenetic regulators [1,7]. They influence DNA methylation and the organisation of higher-order chromatin structures through interactions with other epigenetic modifiers [15]. Epigenetic regulation in vascular and immune cells is also controlled by a network of non-coding RNAs, especially long non-coding RNAs (lncRNAs) [16,17,18,19]. In the context of NCOR1/2, recent studies reveal an intriguing interaction between lncRNAs and their functions, which modulate the stability of repressor complexes and influence signal-dependent gene silencing [7,13,20]. Furthermore, epigenetic regulation and lncRNAs have been recognised as key contributors to vascular pathophysiologies [17,19,21]. Therefore, through epigenetic mechanisms or lncRNAs, NCOR complexes may regulate gene expression by altering chromatin structure and impacting transcription factor activity.

Given its broad roles in inflammation, metabolism, and epigenetics, NCOR1/2 have also been linked to cardiovascular diseases. In atherosclerosis, NCOR1 has been shown to be atheroprotective [9,10,15]. NCOR1 also contributes to vascular injury by maintaining the homeostasis of vascular smooth muscle cells (SMCs) [9]. Little is known about the roles of these metabolic immunomodulators in abdominal aortic aneurysm (AAA). AAA formation involves phenotypic modulation of SMCs and the infiltration of inflammatory cells [22,23,24,25,26,27]. These pathophysiologies, already described in atherosclerosis and cardiovascular diseases, suggest that NCOR1/2 may also play important roles in AAA. Furthermore, epigenetic changes and lncRNAs, which are substantially affected by these immunometabolic regulators, have been recognised as important factors contributing to AAA [28,29,30,31,32]. To date, only two publications have mentioned the role of NCOR1 in AAA [9,22]. Du et al. used a mouse Ang II-induced AAA model and in vitro human SMCs [9]. Chen et al. identified key genes associated with AAA from the Gene Expression Omnibus database, including NCOR1, one of four transcription factors linked to AAA [22]. Regarding NCOR2, nothing is known about its role in AAA.

The aim of this study was therefore to analyse the expression and cellular localisation of NCOR1 and NCOR2 in human AAA tissue and to compare the results with those of the non-aneurysmal aorta from our Swiss Vascular Biobank (SVB) [33], conducting histological and transcriptomics analyses that focus on various markers of vascular and immune cells, as well as on epigenetic factors and lncRNAs.

## 2. Materials and Methods

### 2.1. Tissue Samples

Vascular tissue samples of human aortic tissue used in this study were obtained from patients scheduled for open aortic repair of the infrarenal abdominal aorta (*n* = 60) at the Department of Vascular Surgery (USZ/UZH) and collected in our Swiss Vascular Biobank (SVB). Non-aneurysmal healthy infrarenal aortic samples from the SVB used as reference (*n* = 21) were obtained during kidney transplantation and provided by the Department of Visceral Surgery (USZ/UZH). However, after extraction and determination of RNA quality (RIN, DV200) [33], some samples had to be excluded from this study. As a result, the following number of tissue samples was finally included: AAAs (*n* = 45; 30 elective and 15 ruptured AAAs) and controls (*n* = 18). To estimate the necessary sample size for the current study, we used the already described approaches for transcriptome analyses [34,35,36]. Because almost nothing is known about the expression pattern of NCOR1 and 2 in human AAAs, we used an explorative design of our study for the sample size calculation [35]. Considering two main study groups (AAAs vs. controls) and that the differences between ruptured and elective AAAs might be rather small, we calculated the sample size with a statistical power of 0.8, two-sided significance of 0.05, and a 1.5-fold differences in mRNA expression between the two groups. Consequently, the number of our study samples was adequate.

All patients gave appropriate written informed consent. The local ethics committee (Cantonal Ethics Committee Zurich, Switzerland) approved the tissue sample collection and analysis procedure (BASEC-No 2020-00378 and 2022-00864). The tissue samples were divided for consecutive histological and molecular biological analyses. The part intended for histological studies was fixed in 4% formalin and embedded in paraffin (FFPE). Adjacent tissue pieces used for RNA analyses were collected in a specific cryoprotective solution and stored at −80 °C [29]. This study was conducted in accordance with the World Medical Association Declaration of Helsinki.

### 2.2. Histological Evaluation

To evaluate tissue quality, aneurysm extent, and the composition of healthy aortic samples, FFPE blocks were sectioned into 5 µm slices and stained with Haematoxylin–eosin (HE) and Elastica van Gieson (EvG), focusing on cellular makeup, the extent of inflammation, and the distribution of collagen and elastin fibres. For immunohistochemistry (IHC), FFPE sections were mounted on pre-coated (0.1% poly-L-lysine; Merck/Sigma-Aldrich, Buchs, Switzerland) SuperFrost Plus slides (Thermo Fisher Scientific, Basel, Switzerland), and antigen retrieval was performed by heating in citrate buffer (pH 6.0). The primary antibodies used in this study were sourced from Lucerna-Chem (Luzern, Switzerland) and Agilent/Dako (Basel, Switzerland): anti-nuclear receptor corepressor NCoR1 (PA1-844A, Thermo Fisher/Invitrogen, Basel, Switzerland; abcam ab3482, 1:500), anti-nuclear receptor corepressor NCoR2 (PA1-843, 1:500; Thermo Fisher/Invitrogen), anti-SM-actin (SMCs, abcam ab5694, 1:1000; Lucerna-Chem), anti-CD31 (ECs, ab134168, 1:40; Lucerna-Chem), anti-CD45 (leukocytes, M0701, 1:2000; Agilent/Dako), and anti-CD68 for macrophages (M0814, 1:2000; Agilent/Dako). All antibodies were diluted in DAKO REAL Antibody Diluent (Agilent/Dako). For primary antibody detection, mouse/rabbit-specific HRP/DAB (ABC) detection kits (ab64264, abcam; Lucerna-Chem) and Mayer’s haematoxylin (Carl Roth, Arlesheim, Switzerland) for nuclear counterstaining were used. All slides were scanned and digitised using the Precipoint-M8 Digital Microscope (Formafix, Langenfeld, Germany).

### 2.3. RNA Extraction and Quality Determination

For RNA sequencing and transcriptome analysis, RNA was isolated from fresh-frozen tissue samples using TRIzol (Thermo Fisher), which enables the extraction of total RNA, including non-coding RNAs. The RNA concentration was measured using the NanoDrop Lite Plus Spectrophotometer (Witec, Sursee, Switzerland). To assess the RNA quality of samples suitable for sequencing, RNA integrity numbers (RINs) and the DV200 index (percentage of fragments greater than 200 nucleotides) were determined using TapeStation 4150 (Agilent, Santa Clara, CA, USA). For transcriptome analysis, only samples with sufficient RNA quality—specifically, RIN values between 2 and 4 and a DV200 > 50%—were selected. Such quality standards are typical for atherosclerotic and aneurysmal tissue samples and provide reproducible, reliable data, as described previously [33].

### 2.4. Library Preparation and RNA Sequencing

The RNA library was prepared using the SMARTer Stranded Total RNASeq Kit (Clontech/Takara Bio, San Jose, CA, USA) following the manufacturer’s protocol. The RNA sequencing was conducted in collaboration with the Functional Genomics Center Zurich (FGCZ/ETH, Zürich, Switzerland). To include non-coding RNAs in the analysis, the ribosomal RNA depletion method was employed. The quality and quantity of the RNA library were again validated by determining the RIN. RNA sequencing was performed on the Illumina NovaSeq 6000 (Illumina, Berlin, Germany) with a sequencing depth of 200 M reads across 100 cycles.

### 2.5. Data Analysis

The data analysis of the sequenced RNA was performed using the SUSHI framework developed by FGCZ [34]. The quality control of individual reads was verified by FastQC. Read alignment was carried out with STAR [37,38], and read abundance was estimated using FeatureCounts from the R package (4.5.0) subreads [39]. Differential expression analysis was conducted using the linear model approach from the Bioconductor packages DESeq2 (1.42.0) and EdgeR280 (4.0.16) [40]. The False Discovery Rate (FDR, adjusted *p*-value) was calculated with the Benjamini–Hochberg algorithm for multiple testing [41]. An FDR threshold of <0.1 was considered significant to balance sensitivity and discovery potential within the exploratory framework, recognising the increased likelihood of false positives. The datasets of RNA sequencing generated and analysed during this study are stored on FGCZ servers.

### 2.6. Statistical Analyses

The statistical analyses were conducted using IBM’s SPSS software version 29.0 (SPSS Inc., Chicago, IL, USA). Data were tested for normality using the nonparametric Kolmogorov–Smirnov one-sample test. Due to the broad heterogeneity in individual sample values and the variation in the number of study samples, the Mann–Whitney U test was employed. Correlation analyses were performed using the Spearman correlation coefficient. In all cases, Benjamini–Hochberg correction for multiple testing and adjustments for sex and age were applied [41]. All tests were two-sided, and *p* < 0.05 (*), *p* < 0.01 (**), and *p* < 0.001 (***) were considered statistically significant.

## 3. Results

### 3.1. Patient Characteristics Across Study Groups

The baseline characteristics of the patients included in this study are summarised in Table 1. A total of 45 patients with abdominal aortic aneurysm (AAA) were included: 30 undergoing elective AAA repair (eAAA), 15 with ruptured AAA (rAAA), and 18 healthy aortic controls.

The proportion of male patients was high across all AAA groups (82%), with no significant differences between eAAA, rAAA, and controls. Patients with AAA were significantly older than controls (70.7 ± 10.1 vs. 53.9 ± 6.7 years, *p* < 0.001). Comparing eAAA and rAAA, no statistically significant difference in age was observed. The mean maximal aneurysm diameter was significantly larger in rAAA compared to eAAA (82.2 ± 26.7 mm vs. 63.3 ± 25.2 mm, *p* = 0.017). The prevalence of cardiovascular risk factors and comorbidities in AAA patients was high, with hypertension present in nearly all patients (89%), followed by chronic kidney disease (64%) and coronary heart disease (36%). No significant differences were found between eAAA and rAAA regarding any cardiovascular comorbidities or medication (Table 1). Antiplatelet therapy was the most used medication (76%), followed by statins (51%) and beta-blockers (47%). Overall, the eAAA and rAAA groups were well matched in sex, cardiovascular risk profile, comorbidities, and medications, with the only differences in age and diameter. Concerning healthy aortic controls obtained from organ donors undergoing kidney transplantation, clinical information was limited to age and sex only due to data protection regulations.

### 3.2. Overall Differential Gene Expression Analysis in AAA and Controls

**Figure 1:**
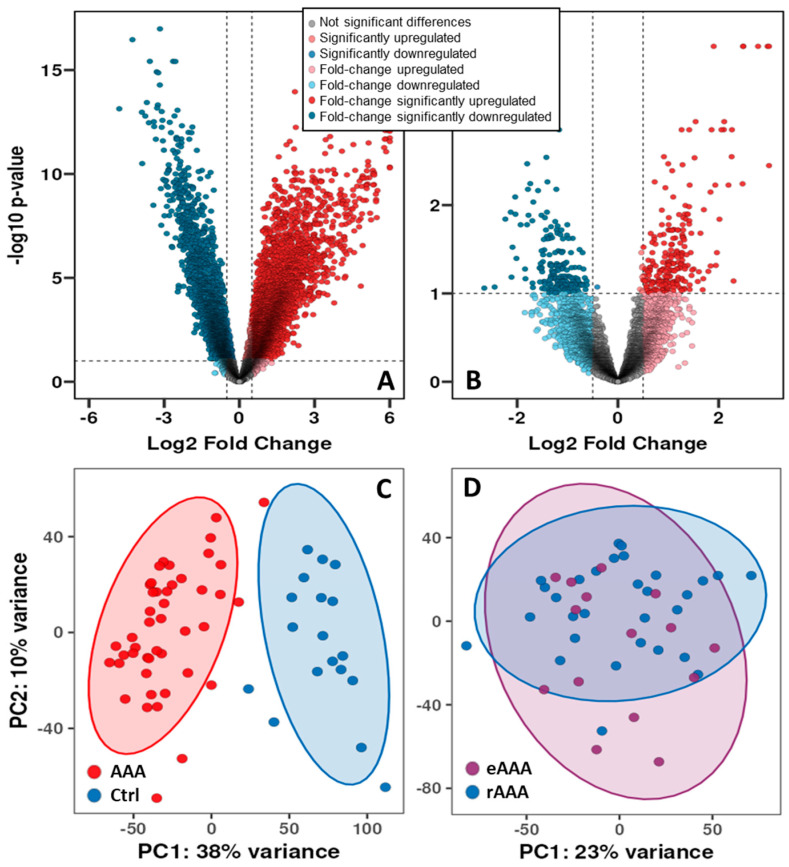
Volcano plot showing differentially expressed genes in the abdominal aortic aneurysm (AAA) samples compared to control healthy aorta (Ctrl) (**A**) and between elective (eAAA) and ruptured (rAAA) aneurysm samples (**B**). Principal component analysis (PCA) of the study groups comparing the main principal component (PC) variances PC1 and PC2, (**C**) AAA against Ctrl, (**D**) eAAA vs. rAAA. The volcano plot shows −log10-transformed FDR as a function of the difference between the study groups. The broken lines indicate a 0.5 log2-fold change with FDR < 0.1. Fold-change means x-times increase or decrease in expression.

To assess overall transcriptional expression patterns, differential gene expression analysis was performed comparing RNA sequencing results from AAA samples, non-aneurysmal control aortas, and elective versus ruptured AAAs. Volcano plot analysis demonstrated significant transcriptional changes in many genes in AAA compared to controls, exceeding the predefined threshold of log2 fold change ≥ 0.5 and FDR < 0.1 (Figure 1A). In AAA, 5552 genes were upregulated, while 3063 were downregulated.

Of these genes, 2264 were lncRNAs (1435 with unknown functions or not yet characterised). The principal component analysis (PCA) (Figure 1C) shows a clear separation between the groups (AAA, control).

In contrast, comparison of eAAA and rAAA revealed a significantly smaller set of differentially expressed genes (Figure 1B), with 238 upregulated and 183 downregulated (including 78 lncRNAs). This suggests that, while aneurysm formation involves broad transcriptomic changes, rupture is characterised by more subtle differences in gene expression. The sets of differentially expressed genes were linked to extracellular matrix degradation, remodelling, and SMC phenotypic switching (various proteolytic enzymes, collagen fibres, markers of synthetic smooth muscle cells), as well as inflammation (including various cytokines, including T-cells, macrophages and others). Furthermore, the PCA showed considerable overlap and no clear separation of individual samples between eAAA and rAAA (Figure 1D).

### 3.3. Differential Expression Analysis of NCOR1 and NCOR2 in AAA and Controls

**Figure 2:**
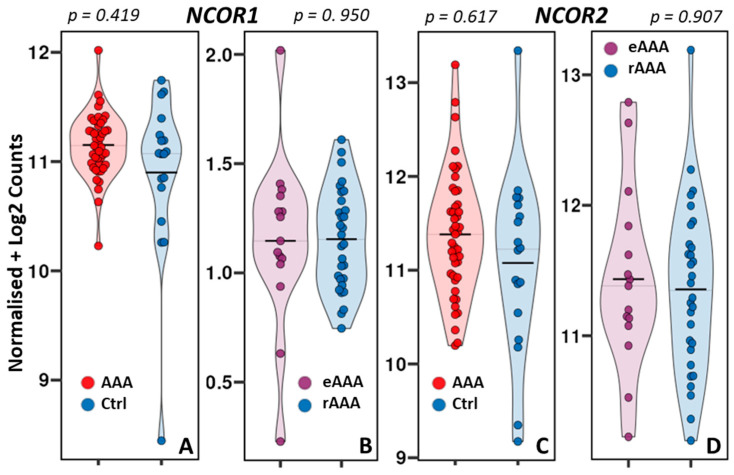
Violin plots comparing the expression of *NCOR1* (**A**,**C**) and *NCOR2* (**B**,**D**) in abdominal aortic aneurysm (AAA) against healthy control aorta (Ctrl) (**A**,**B**) and between elective (eAAA) and ruptured AAA (rAAA) (**C,D**). The width of each violin reflects the data density, and the median is indicated by the central line. Each point represents an individual sample.

Given the scope of this study, the differential expression analysis concentrated on *NCOR1* and *NCOR2*. Surprisingly, in contrast to previous research on human carotid plaques showing significantly reduced *NCOR1* expression in carotid atherosclerotic lesions [10], violin plot analysis revealed no significant difference in *NCOR1* expression between AAA and the control aorta (*p* = 0.419) (Figure 2A). Similarly, *NCOR2* expression did not differ significantly between the two study groups (*p* = 0.617) (Figure 2B). Furthermore, when comparing elective and ruptured aneurysms, the expression patterns for *NCOR1* and *NCOR2* were almost identical (*p* = 0.950 and *p* = 0.907) (Figure 2C,D).

Based on the results shown above, which indicate no differences between eAAA and rAAA in the expression of *NCOR1* and *NCOR2* (as discussed in the Discussion, paragraph Study limitations), we focused on only two study groups in the further analyses: AAA and healthy aortic controls.

### 3.4. Histological Analysis of NCOR1 and NCOR2 in AAA and Controls

**Figure 3:**
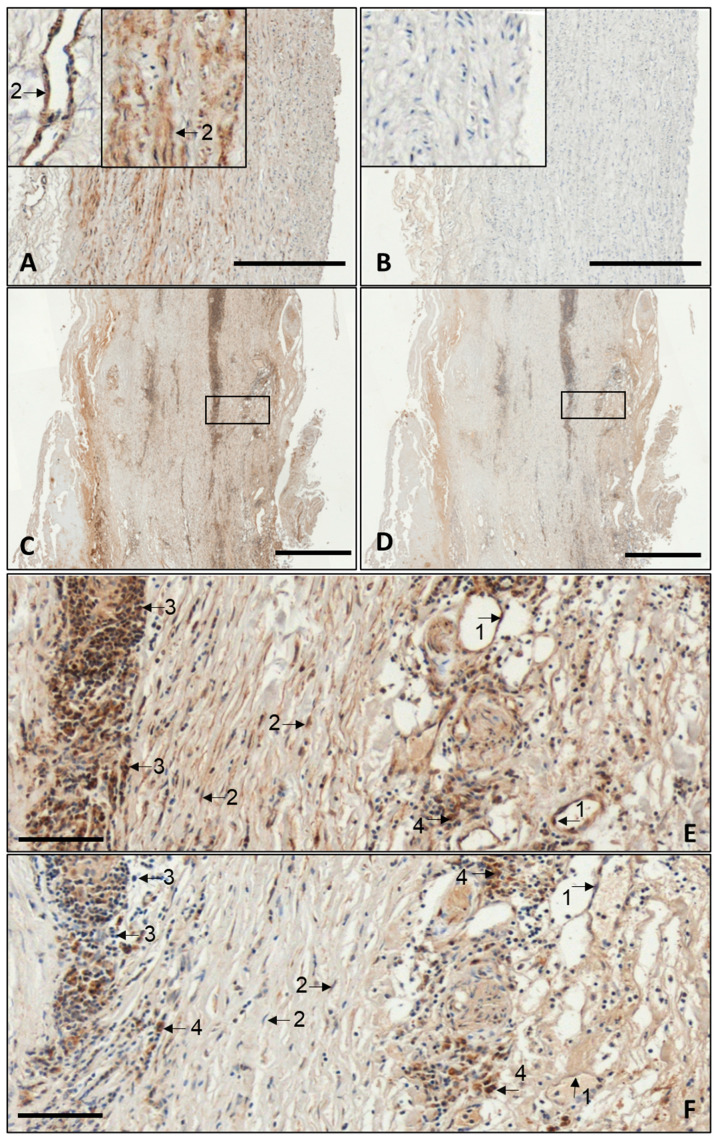
Representative images of histological analysis of NOCR1 and 2 in human AAA tissue samples and aortic controls (Ctrl). The images (**A**–**D**) show overview stains of control aorta with NCOR1 (**A**) and NCOR2 (**B**), as well as AAA tissue stained with NCOR1 (**C**) and NCOR2 (**D**). Images (**E**,**F**) provide more detailed insight into the expression of NCOR1 and NCOR2 in individual cells within the AAA wall: (**E**) NCOR1; (**F**) NCOR2. Arrows: endothelial cells (ECs) (1), smooth muscle cells (SMCs) (2), leukocytes CD45 (3), and macrophages (CD68) (4). Scale bars represent 500 μm on (**A**–**D**), 100 μm on (**E**,**F**). AAA: abdominal aortic aneurysm.

To assess the cellular localisation of NCOR1 and NCOR2 expression, corresponding immunohistochemistry (IHC) was performed (Figure 3). Again, also in histology, no significant differences were observed between eAAA and rAAA. Therefore, Figure 3 displays only representative images of a healthy control aorta and AAA in general. In the controls, NCOR1 expression was detected solely in smooth muscle cells (SMCs) within the media and in pericytes surrounding larger vessels in the adventitia (Figure 3A). Regarding NCOR2, no expression was observed in healthy aortas (Figure 3B). In contrast, multiple cell types were found to be positive for both immunometabolic factors in aneurysmal tissue (Figure 3C–F). NCOR1 was positive in ECs, SMCs, CD45, and CD68 cells (Figure 3E). In contrast, NCOR2 was only detected in macrophages (CD68) (Figure 3F).

### 3.5. Correlation Analysis of NCOR1 and NCOR2 with Factors Associated with AAA

Since IHC cannot capture all cellular features within AAA, correlation analyses of *NCOR1* and *NCOR2* expression with known markers of ECs, SMCs, immune cells, and neovascularisation were conducted (Table 2 and Table 3). Additionally, correlation analyses were performed for other aneurysm-associated factors, including proteolytic enzymes, epigenetic factors, and lncRNAs (Table 3 and Table 4). As already mentioned in the Section 2, to minimise potential bias and false-positive or false-negative correlations, Benjamini–Hochberg correction for multiple tests and adjustment for age and sex were performed.

Association with smooth muscle cell markers. The *NCOR1* expression in healthy aortas was positively correlated with *SMTN*, a contractile marker of SMCs (*r* = 0.618, *p* < 0.05), as well as with the expression of some extracellular matrix components such as FN1, *FBN1*, and the factor *PDGFRB* (*r* = 0.519, 0.581, 0.616, *p* < 0.05) (Table 2), along with collagen *COL1A1* and *COL3A1* (*r* = 0.496 and 0.542, *p* < 0.05). Conversely, in AAA tissue samples, NCOR1 exhibited significant inverse correlations with multiple SMC genes, including both contractile and synthetic phenotypes (*ACTA2, SMTN, FN1, COL1A1, COL3A1*, *FBN1*, and *PDGFRB*; *r* = −0.373 to −0.572, *p* < 0.05, 0.01, and <0.001) (Table 2). Interestingly, *NCOR2* did not show any significant associations with SMC markers in either healthy aortas or AAA tissue, suggesting a potential lack of its effect in SMCs compared to *NCOR1*.

Association with endothelial markers. Neither *NCOR1* nor *NCOR2* expression correlated with endothelial cell (EC) markers in the intact healthy aorta (Table 2). In contrast, in AAA tissue samples, *NCOR1* showed an inverse correlation with several EC- and angiogenesis-related genes. Notably, higher *NCOR1* expression was associated with lower *CDH5* (*VE*-cadherin) (*r* = −0.34, *p* < 0.05), as well as reduced expression of the angiogenic factor *TIE1* (*r* = −0.349, *p* < 0.05) and *ANG* (*r* = −0.367, *p* < 0.05). This suggests that *NCOR1* may be downregulated in endothelial- and neovessel-rich areas of AAA. *NCOR2* showed a different pattern, being strongly negatively associated with the expression of the EC-marker *VCAM1* (*r* = −0.464, *p* < 0.001). Furthermore, *NCOR2* correlated positively with *VEGFB* (*r* = 0.660, *p* < 0.001), indicating that the effects of *NCOR1* and *NCOR2* differ in neovascularisation.

Association with immune cell markers. In AAA tissue samples, *NCOR1* expression showed significant positive correlations with certain T-lymphocyte markers (Table 3). Specifically, *NCOR1* correlated with *CD3D, CD3E, CD3G, and CD247* (*r* = 0.325 to 0.412, *p* < 0.05). *NCOR1* also weakly correlated with the T-cell chemokine *CXCL9* (*r* = 0.384, *p* < 0.05), indicating a link between *NCOR1* and T cells. Additionally, NCOR1 exhibited a strong positive correlation with the apoptotic caspase *CASP8* (*r* = 0.555, *p* < 0.001). In contrast, *NCOR2* showed a more limited immune profile, with no significant associations with T-cell markers. Instead, *NCOR2* was positively correlated with *NFκB2* (*r* = 0.519, *p* < 0.01) and *TGFB1* (*r* = 0.632, *p* < 0.001). Notably, *NCOR2* was significantly inversely associated with *CXCL10* (*r* = −0.505, *p* < 0.01).

Association with ECM degradation enzymes. Striking differences were also observed in how *NCOR1* and *NCOR2* relate to proteolytic enzymes in AAA. *NCOR1* was significantly inversely correlated with many matrix-degrading proteases (Table 3). Higher *NCOR1* expression was significantly associated with lower levels of *MMP2* (*r* = −0.445, *p* < 0.01) and *MMP14* (*MT1-MMP*; *r* = −0.649, *p* < 0.001), key metallo-proteinases that drive elastic and collagen fibre degradation. Similarly, *NCOR1* showed significant negative correlations with *ADAM15* (*r* = −0.417, *p* < 0.05), and ADAMTS family proteases involved in collagen and elastin cleavage, including *ADAMTS2* (*r* = −0.473, *p* < 0.01) and *ADAMTS7* (*r* = −0.499, *p* < 0.01). In contrast, *NCOR2* showed positive correlations with *ADAMTS2* (*r* = 0.441, *p* < 0.01) and *ADAMTS7* (*r* = 0.476, *p* < 0.01), suggesting a link to reparative vascular remodelling.

Associations with epigenetic factors. AAA tissues showed numerous intriguing correlations between *NCOR1/2* and many epigenome-regulating genes (Table 4). For example, *NCOR1* levels were significantly associated with several histone methyltransferases (HMTs), including *KMT2A, KMT2C, EHMT1, EZH1, EZH2,* and *KMT3*, as well as numerous histone demethylases (HDMs), including *KDM2A/B, KDM3B, KDM4A/C, KDM5A/D*, and *KDM8A*. Similarly, *NCOR1* was significantly positively associated with several histone acetyltransferases, including *KAT6A/B* and *KAT8*, as well as histone deacetylases *HDAC1* and *HDAC6*. Moreover, *NCOR1* also showed a positive correlation with DNA methyltransferase *DNMT1*. These widespread positive correlations indicate a strong link between *NCOR1* and epigenetic enzymes in AAA.

In contrast to *NCOR1*, *NCOR2* exhibited an almost reciprocal pattern for many epigenetic factors (Table 4). *NCOR2* expression was positively correlated with histone methyltransferases (HMTs) similar to *NCOR1*, but interestingly, with different ones (KMT2D, *SETD1A/B*, and *KMT5C*). Surprisingly, histone demethylases (HDMs) showed negative correlations with *NCOR2, KDM1B, KDM3A, KDM4C, KDM5A*, and *KDM6A*. Regarding histone deacetylases (HDACs), the correlations were positive for *HDAC4, 5*, and *7*, and negative for *HDAC2* (Table 4). Interestingly, unlike *NCOR1, NCOR2* showed a significant correlation with the DNA methyltransferase *DNMT3A*. Additionally, mixed results were observed for the sirtuin deacetylases, another important class of epigenetic regulators. While *NCOR1* showed no correlations with sirtuins, *NCOR2* exhibited a negative association with SIRT1 and a significant positive association with *SIRT2* and *SIRT6*.

These data suggest that in AAA, NCOR1 and NCOR2 are important epigenetic modulators of disease progression, operating, however, within different epigenetic contexts.

Associations with lncRNAs. The correlation analysis of *NCOR1* and *NCOR2* with lncRNAs also reflected the differences between these two immunometabolic modulators. *NCOR1* expression was strongly positively correlated with the pro-proliferative lncRNA *MALAT1* (*r* = 0.485, *p* < 0.01). *NCOR2*, on the other hand, showed strong positive correlations with *H19* (*r* = 0.620, *p* < 0.001) and was negatively associated with the senescence-associated lncRNA GAS5 (*r* = −0.437, *p* < 0.01).

## 4. Discussion

Interestingly, in contrast to previous data from human atherosclerotic carotid lesions [10,15], our transcriptomics analyses revealed no significant differences in the expression of NCOR1 or NCOR2 between AAA and control aortas. However, significant divergencies were observed at the cellular level. While NCOR1 was broadly detectable in the AAA tissue across endothelial, smooth muscle, and inflammatory cells, NCOR2 exhibited a more restricted pattern, predominantly associated with macrophage-rich areas. Correlation profiling further linked *NCOR1* and *NCOR2* to distinct signatures in terms of their cellular association, extracellular matrix remodelling, epigenetic regulation, and lncRNAs, supporting non-redundant roles for both corepressors in AAA.

Unlike previous observations in human carotid atherosclerotic plaques, where NCOR1 expression was significantly reduced compared to controls [10,15], our transcriptomic data showed no significant difference in *NCOR1* or *NCOR2* expression between AAA and the healthy aorta. In this context, we suggest that disease-specific factors distinguish AAA from atherosclerosis. In atherosclerotic lesions, NCOR1 has been linked to macrophages and foam cell-rich plaques, where it reduces lipid uptake pathways [10]. In contrast, carotid atherosclerosis is primarily an intimal disease, whereas AAA affects all layers of the aortic wall and is characterised by extensive matrix remodelling rather than the formation of a lipid-rich core [23,27,42]. Moreover, although macrophages are present in AAA tissue, our previous study [26] demonstrated that lymphocytes, including B and T cells, predominate with the inflammatory infiltrates, whereas advanced atherosclerotic plaques are typically characterised by macrophage-rich lesions [15,42,43]. Our findings suggest that in AAA, downregulation of *NCOR1* in one cell type (e.g., dedifferentiating SMCs) may be counterbalanced by upregulation in other cell types (e.g., inflammatory cells), resulting in cell-specific alterations without detectable changes at the tissue level. This assumption is supported by a recent study using a mouse AAA model, which showed that NCOR1 expression can increase in aneurysmal aortas as a compensatory response [9]. Therefore, the absence of significant mRNA differences between AAA and healthy aorta does not indicate a lack of involvement of NCORs in AAA but rather highlights their context- and cell-type-dependent regulation.

Despite minimal transcriptional differences, distinct localisation patterns of NCOR1 and NCOR2 were observed at the protein level. NCOR1 shows broad expression across multiple cell types in AAA, whereas NCOR2 appears to be predominantly expressed in immune cells, particularly macrophages. These findings are consistent with previous mechanistic studies emphasising cell-type-specific utilisation of NCOR complexes [5]. A prior study demonstrated that SMC-specific NCOR1 is essential for maintaining the contractile phenotype and vessel wall integrity [9]. In contrast, NCOR2 was not detected in SMCs of AAA lesions. Instead, its prominent presence in macrophages supports its established role as an immunomodulatory corepressor in myeloid cells [10,11,12].

The extended correlation analyses discussed below provide further insight into the potential roles of *NCOR1* and *NCOR2* in AAA pathogenesis. However, it is important to emphasise that despite correction for multiple testing and adjustment for age and sex, our interpretations remain associative, as correlation does not imply causation.

NCOR1 expression showed positive correlations with multiple SMC markers, including SMTN, as well as with various extracellular matrix (ECM) components produced by these cells. These data indicate that in the healthy aortic wall, higher *NCOR1* levels are associated with a contractile phenotype and increased synthesis of structural proteins that stabilise the vessel wall [9]. In contrast, in AAA tissue, *NCOR1* expression was inversely correlated with numerous SMC-related genes, including both contractile markers (*ACTA2, SMTN*) and synthetic/ECM genes (*FN1, COL1A1, COL3A1, FBN1*), suggesting that SMCs within the aneurysmal wall have lost a substantial portion of *NCOR1* expression. Du et al. reported that loss of NCOR1 drives SMCs towards a “dedifferentiated” state associated with increased MMP production and AAA formation [9]. Thus, our human data suggest that SMCs in AAA tissue exhibit reduced *NCOR1* expression, consistent with *NCOR1* downregulation as a feature of pathological remodelling. The opposing pattern observed between healthy and aneurysmal tissue further indicated a redistribution of NCOR1 away from SMCs during AAA development, potentially contributing to SMC dysfunction. These findings are supported by the downregulation of *PDGFRB* alongside *NCOR1* in SMCs, reflecting altered phenotypic switching and loss of contractile stability [9,24]. In contrast to *NCOR1, NCOR2* showed no association with SMC signatures in either healthy or diseased aorta.

Regarding endothelium, neither *NCOR1* nor *NCOR2* showed notable correlations with ECs or angiogenic markers. However, in AAA samples, *NCOR1* expression was inversely correlated with *VE*-cadherin (*CDH5*), as well as with TIE1 receptor and angiopoietin levels. These findings are consistent with a recent mechanistic study demonstrating that NCOR1 can influence angiogenesis [14]. Our data therefore suggest that in AAA, NCOR1 may facilitate neovascularisation. In contrast, *NCOR2* levels correlated positively with *VEGFB*, suggesting a role in maintaining existing vascular structures. These findings further support distinct functional roles of NCOR1 and NCOR2 in vascular biology.

Chronic inflammation is a central feature of AAA development [23,27]. Our data indicate that NCOR1 and NCOR2 are associated with distinct immune signatures. In aneurysmal tissue, *NCOR1* expression showed significant positive correlations with various T-lymphocyte markers and cytokine signalling pathways. These findings are somewhat unexpected, given that NCOR1 has been reported to suppress gene expression in macrophages [10,12,44]. However, in contrast to atherosclerosis, AAA is characterised by a predominance of T cells rather than macrophages [26]. Furthermore, it has been reported that in resting macrophages, NCOR1 represses certain pro-inflammatory genes, whereas its loss leads to hyperactivation of these pathways [45]. We also observed a strong positive correlation between *NCOR1* and an apoptotic caspase *CASP8*, suggesting its potential role in active cell death. These findings suggest that NCOR1, although generally anti-inflammatory at the cellular level, may be recruited to areas of active immune responses to modulate inflammation and limit tissue damage [10]. In contrast, *NCOR2* displayed a more selective and partially inverse relationship with immune markers. *NCOR2* showed strong positive correlations with *TGF-β1* and *NF-κB2*. *TGF-β1* is known to be upregulated in AAA during tissue remodelling [46]. The strong association between *NCOR2* and *TGF-β1* suggests a role in immunomodulation and repair processes. The positive correlation with *NF-κB2* may indicate involvement in the non-canonical NF-κB signalling. Additionally, *NCOR2* was inversely correlated with *CXCL10*, a chemokine involved in Th1-type T-cell recruitment [44,47], consistent with the established role of NCOR2 (SMRT) in restraining pro-inflammatory transcriptional activation [1,12,20]. Collectively, these findings indicated that NCOR1 and NCOR2 operate in distinct inflammatory contexts within AAA, with NCOR1 being associated with active inflammation and apoptosis [4,10,15] and NCOR2 with regulatory and remodelling processes [1].

Further correlation analyses indicated that NCOR1 and NCOR2 are also differentially associated with ECM degradation. *NCOR1* expression was significantly negatively correlated with multiple proteolytic enzymes, including *MMP2*, *MMP14*, *ADAM15*, *ADAMTS2*, and *ADAMTS7*, suggesting a protective role in limiting ECM degradation and maintaining aortic wall integrity [9]. In contrast, *NCOR2* showed positive correlations with *ADAMTS2* and *ADAMTS7*. While *ADAMTS2* contributes to collagen maturation [48], ADAMTS7 has been implicated in SMC migration and vascular remodelling [49]. These findings suggest that NCOR2 might support control of targeted vascular remodelling.

As transcriptional corepressors, NCOR1 and NCOR2 also play a key role in epigenetic regulation of gene expression [3,4,7,15]. This was confirmed by our correlation analysis. *NCOR1* expression showed positive correlation with a wide range of epigenetic regulators, including HMTs, HDMs, HATs, and HDACs. Additionally, *NCOR1* levels were associated with increased expression of the DNA methyltransferase *DNMT1*. These findings imply a significant role for NCOR1 in chromatin reprogramming at multiple levels, including SMC phenotype switching and activation/repression of specific inflammatory cytokines or cells [9]. The positive correlations may reflect a stress response programme triggered by injury or inflammation within the diseased aortic wall. In contrast, *NCOR2* showed a more selective epigenetic profile. *NCOR2* also correlated positively with HMTs, but with different ones than those linked to *NCOR1*. Moreover, *NCOR2* was negatively associated with HDMs. These results suggest that NCOR2 is involved in gene silencing and transcriptional repression [1]. The correlation with HDACs also involved different deacetylases than for NCOR1. Furthermore, as previously described, NCOR2 appears to support NCOR1 in the epigenetic regulation [12,20]. Additionally, *NCOR1* and *NCOR2* were associated with sirtuins *SIRT1*, *2*, and *6*. Sirtuins are a specific class of histone deacetylases regulating the acetylation in an ATP-independent manner, using NAD+ as a cofactor, and thus linking their activity to redox balance, energy availability, and stress signalling [50,51]. In summary, NCOR1 and 2 seem to have a broad epigenetic reprogramming signature, contributing to distinct yet interconnected epigenetic networks in AAA.

Long non-coding RNAs (lncRNAs) have already been recognised as essential regulators in cardiovascular diseases, including AAA [16,19,28,30]. Our study revealed distinct correlations between *NCOR1/2* and specific lncRNAs, suggesting their involvement in lncRNA-regulated networks in AAA. *NCOR1* expression was strongly positively correlated with *MALAT1*, whereas *NCOR2* showed positive correlation with *H19* and negative association with *GAS5*. *MALAT1* (Metastasis-Associated Lung Adenocarcinoma Transcript 1) has been linked to endothelial proliferation, angiogenesis, and SMC migration [18,52]. *H19* is upregulated in both human and experimental AAAs and has been associated with aneurysm progression [29,49]. Our data indicate that *NCOR2* expression is associated with elevated *H19* levels. One possible explanation could involve the SMC phenotype, as *H19* has been linked to SMC apoptosis and loss in AAA [29]. It is also plausible that *H19* may regulate *NCOR2* expression. *GAS5* (Growth Arrest Specific 5) inversely correlates with *NCOR2* and has been reported to promote AAA and SMC apoptosis [32]. Lower *GAS5* levels in areas with high *NCOR2* expression suggest that cells in those areas may be active or proliferating. This aligns with the observation that NCOR2 is present in macrophages engaged in vascular remodelling. These distinct associations further support the assumption that NCOR1/2 operate within separate regulatory networks in AAA, involving SMC physiology, immune response, and epigenetics. It is also conceivable that lncRNAs may modulate NCOR complexes, thereby influencing their expression.

Our findings have several potential therapeutic implications for AAA and related vascular diseases. First, NCOR1 appears to act as a protective factor in the vessel wall, particularly by supporting SMC stability and suppressing ECM degradation. Increasing NCOR1 activity in SMCs might offer a new strategy to reinforce the aortic wall against aneurysmal dilatation. Agonists or modulators that promote NCOR1 recruitment could indirectly enhance its positive effect in AAA pathophysiology [10]. Conversely, NCOR2 could be targeted to reduce immune-driven damage in AAA. Targeting NCOR1/2 and their associated regulatory pathways may provide combined benefits, including reduced inflammation, improved SMC function, and restoration of epigenetic balance within the diseased aorta.

Study limitations. This study has several notable limitations. First, the sample size and heterogeneity. While we analysed a moderate study cohort (45 AAA and 18 controls), human AAA tissues are highly variable in composition, which can confound the transcriptomic analyses. Furthermore, we did not observe any differences between eAAA and rAAA. To properly compare these two disease conditions, the tissue samples of patients with rAAA had to be removed at the site of rupture. This, however, is often not possible, and excision farther away from the ruptured site might not reflect the aortic wall prone to rupture. Regarding the correlation analyses, some of our observed associations might be affected by outliers or specific sub-regions of the aneurysm wall. Furthermore, the study is observational by design. Therefore, we cannot establish causality from the ascertained correlations. As a result, our interpretations are speculative, as correlation does not imply causation. Nonetheless, we provide potential explanations and relevant literature supporting our assumptions. In addition, control aorta samples were obtained from organ donors of a younger age. The significant age difference between controls and AAA patients could be an additional potential confounder. It is worth mentioning that obtaining a healthy aorta of a matched AAA cohort of 70 years on average is almost impossible. In summary, the findings of our current study should be regarded as hypothesis-generating.

## 5. Conclusions

Despite the above-described limitations, our study provides, for the first time, a comprehensive view of NCOR1 and NCOR2 in human AAA, revealing distinct expression patterns, cellular localisations, and molecular associations that suggest non-redundant roles in the underlying disease. Our data align with findings from related vascular pathologies but also raise new questions, particularly about NCOR2’s function in AAA. However, because our data on the role of these nuclear receptors in human AAA are not conclusive, more nuanced approaches are necessary, such as spatial transcriptomics or single-cell RNA sequencing, to assess, for instance, NCOR1/2 activity in specific cell subpopulations within the diseased aortic wall. Clarifying these roles could provide a basis for developing new diagnostic markers and innovative therapeutic targets for AAA and possibly other chronic vascular diseases.

## Figures and Tables

**Table 1:** Patient characteristics.

	AAA	eAAA	rAAA	Ctrl	*p*-Values
	(*n* = 45)	(*n* = 30)	(*n* = 15)	(*n* = 18)	
Sex (m/f %)	37 (82%)	26 (87%)	11 (73%)	12 (80%)	0.243
Age (years)	70.7 ± 10.1	68.6 ± 10.9	74.9 ± 6.6	53.9 ± 6.7	0.043 (<0.001) **
AAA diameter (mm)	69.7 ± 26.9	63.3 ± 25.2	82.2 ± 26.7	-	0.017 *
Comorbidities					
Hypertension	40 (89%)	27 (90%)	13 (87%)	-	1.000
Hyperlipidaemia	16 (36%)	12 (40%)	4 (27%)	-	0.514
Nicotine abuse	17 (38%)	12 (40%)	5 (33%)	-	0.752
Diabetes mellitus	6 (13%)	3 (10%)	3 (20%)	-	0.384
Chronic kidney disease	29 (64%)	19 (63%)	10 (67%)	-	1.000
Coronary heart disease	16 (36%)	12 (40%)	4 (27%)	-	0.514
Peripheral artery disease	7 (16%)	5 (17%)	2 (13%)	-	1.000
Stroke	4 (9%)	3 (10%)	1 (7%)		1.000
Medication					
Antiplatelet therapy	34 (76%)	24 (80%)	10 (67%)	-	1.000
Alpha-blocker	4 (9%)	4 (13%)	-	-	0.285
Beta-blocker	21 (47%)	14 (47%)	7 (47%)	-	0.530
ACE inhibitors	12 (27%)	10 (33%)	2 (13%)	-	0.283
AT-blocker	5 (11%)	3 (10%)	2 (13%)	-	0.613
Statins	23 (51%)	16 (53%)	7 (47%)	-	1.000
Diuretics	7 (16%)	6 (20%)	1 (7%)	-	0.395

Age and AAA diameter were normally distributed; therefore, a parametric *t*-test was used. For all other values, Fisher’s exact test was applied. * *p*-values comparing eAAA vs. rAAA; ** *p*-values in brackets show statistical comparison between AAA and Ctrl. AAA: abdominal aortic aneurysm, eAAA: elective AAA, rAAA: ruptured AAA, Ctrl: healthy aortic controls, m: male, f: female, ACE: angiotensin-converting enzyme, AT: angiogensin

**Table 2:** Correlations between *NCOR1* and *2* and various markers of SMCs and ECs.

	Contractile SMCs		Synthetic SMCs				
	*ACTA2*	*SMTN*	*FN1*	*COL1A1*	*COL3A1*	*FBN1*	*PDGFRB*
	Non-aneurysmal healthy aorta (Ctrl)						
*NCOR1*	n.s.	0.618 *	0.581 *	0.542 *	0.496 *	0.519 *	0.616 *
*NCOR2*	n.s.	n.s.	n.s.	n.s.	n.s.	n.s.	n.s.
	Abdominal aortic aneurysm (AAA)						
*NCOR1*	−0.418 *	−0.373 *	−0.447 **	−0.572 ***	−0.547 ***	−0.433 *	−0.504 ***
*NCOR2*	n.s.	n.s.	n.s.	n.s.	n.s.	n.s.	n.s.
	ECs		Neovascularisation				
	*CDH5*	*VCAM1*	*VEGFB*	*TIE1*	*ANG*		
	Non-aneurysmal healthy aorta (Ctrl)						
*NCOR1*	n.s.	n.s.	n.s.	n.s.	n.s.		
*NCOR2*	n.s.	n.s.	n.s.	n.s.	n.s.		
	Abdominal aortic aneurysm (AAA)						
*NCOR1*	−0.340 *	n.s.	n.s.	−0.349 *	−0.367 *		
*NCOR2*	n.s.	−0.464 ***	0.660 ***	n.s.	n.s.		

Spearman correlation coefficient. Benjamini–Hochberg (B-H) correction for multiple variables and adjustment for sex and age were applied to determine statistical significance: * *p* < 0.05, ** *p* < 0.01, *** *p* < 0.001; n.s. not significant. ECs: endothelial cells, SMCs: smooth muscle cells; *NCOR1* and *2*: nuclear receptor co-repressor 1 and 2; *ACTA2*: alpha-smooth muscle actin, *SMTN*: smoothelin, *FN1*: fibronectin, *COL1A1*: collagen 1, *COL3A1*: collagen 3, *FBN1*: fibrillin-1, *PDGFRB*: platelet-derived growth factor receptor beta, *CDH5*: *VE*-cadherin, *VCAM1*: vascular cell adhesion molecule 1, *TIE1*: tyrosine kinase with immunoglobulin-like domain 1, *VEGFB*: vascular endothelial growth factor B, *ANG*: angiopoietin.

**Table 3:** Correlations between *NCOR1/2* and various markers of inflammation and ECM degradation in AAA.

	Inflammatory Cells								Apoptosis
	*CD3D*	*CD3E*	*CD3G*	*CD247*	*NFKB2*	*TGFB1*	*CXCL9*	*CXCL10*	*CASP8*
*NCOR1*	0.325 *	0.371 *	0.412 *	0.375 *	n.s.	n.s.	0.384 *	n.s.	0.555 ***
*NCOR2*	n.s.	n.s.	n.s.	n.s.	0.519 **	0.632 ***	n.s.	−0.505 **	n.s.
	Proteolytic enzymes/ECM degradation								
	*MMP2*	*MMP14*	*ADAM15*	*ADAMTS2*	*ADAMTS7*				
*NCOR1*	−0.445 **	−0.649 ***	−0.417 *	−0.473 **	−0.499 **				
*NCOR2*	n.s.	n.s.	n.s.	0.441 **	0.476 **				

Spearman correlation coefficient. Benjamini–Hochberg (B-H) correction for multiple variables and adjustment for sex and age were applied to determine statistical significance: * *p* < 0.05, ** *p* < 0.01, *** *p* < 0.001; n.s. not significant. *NCOR1* and *2*: nuclear receptor co-repressor 1 and 2, *CD3D*, *CD3E*, *CD3G*, *CD247*: T-cell surface glycoproteins, *NFKB2*: Nuclear factor NF-kappa-B p100 subunit, *TGFB1*: Transforming growth factor beta 1, *CXCL9* and *10*: Chemokine (C-X-C motif) ligand 9 and 10, *CASP8*: Apoptosis marker caspase 8, *MMP*: matrix metalloproteinase, *ADAM*: A disintegrin and metalloproteinase, *ADAMTS*: A disintegrin and metalloproteinase with thrombospondin motifs.

**Table 4:** Correlations between *NCOR1* and *2* and various markers of epigenetics and lncRNAs in AAA.

	HMTs											
	*KMT2A*	*KMT2C*	*KMT2D*	*SETD1A*	*SETD1B*	*EHMT1*	*EZH2*	*KMT3A*	*KMT5C*			
*NCOR1*	0.486 **	0.525 **	n.s.	n.s.	n.s.	0.468 **	0.465 **	0.537 **	n.s.			
*NCOR2*	n.s.	n.s.	0.642 ***	0.628 ***	0.653 ***	n.s.	n.s.	n.s.	0.476 **			
	HDMs											
	*KDM1B*	*KDM2A*	*KDM2B*	*KDM3A*	*KDM3B*	*KDM4A*	*KDM4C*	*KDM5A*	*KDM5D*	*KDM6A*	*KDM7A*	*KDM8*
*NCOR1*	n.s.	0.452 **	0.477 **	n.s.	0.446 **	0.392 *	0.555 ***	0.487 **	0.441 **	n.s.	n.s.	0.532 **
*NCOR2*	−0.447 **	n.s.	n.s.	−0.453 **	n.s.	n.s.	−0.464 **	−0.556 ***	n.s.	−0.487 **	−0.403 *	n.s.
	HATs			HDACs								
	*KAT6A*	*KAT6B*	*KAT8*	*HDAC1*	*HDAC2*	*HDAC4*	*HDAC5*	*HDAC6*	*HDAC7*			
*NCOR1*	0.469 **	0.419 *	0.401 *	0.451 **	n.s.	n.s.	n.s.	0.447 **	n.s.			
*NCOR2*	n.s.	n.s.	n.s.	n.s.	−0.441 **	0.467 **	0.709 ***	n.s.	0.490 **			
	DNMTs		Sirtuins				lncRNAs					
	*DNMT1*	*DNMT3A*	*SIRT1*	*SIRT2*	*SIRT6*		*H19*	*MALAT1*	*GAS5*			
*NCOR1*	0.449 **	n.s.	n.s.	n.s.	n.s.		n.s.	0.485 **	n.s.			
*NCOR2*	n.s.	0.453 **	−0.511 **	0.452 **	0.589 ***		0.620 ***	n.s.	−0.437 **			

Spearman correlation coefficient. Benjamini–Hochberg (B-H) correction for multiple variables and adjustment for sex and age were applied to determine statistical significance: * *p* < 0.05, ** *p* < 0.01, *** *p* < 0.001; n.s. not significant. *NCOR1* and *2*: nuclear receptor co-repressor 1 and 2, HMTs: histone methyltransferases KMT: lysine (K) histone methyltransferase, HDMs: histone demethylases, KDM: lysine (K) histone demethylase, HATs: histone acetyl transferases, KAT: lysine (K) histone acetyl transferase; SIRT1, 2, 6: sirtuins, lncRNAs: long non-coding RNAs. *MALAT1*: lncRNA Metastasis-Associated Lung Adenocarcinoma Transcript 1, *GAS5*: lncRNA Growth Arrest Specific 5.

## Data Availability

All data used in this study are stored on the servers of FGCZ. Datasets used in the current publication are stored at http://www.ncbi.nlm.nih.gov/bioproject/1454268 (accessed on 14 April 2026).

## Abbreviations

NCOR1: Nuclear receptor corepressor 1; NCOR2: Nuclear receptor corepressor 2; SMRT: silencing mediator of retinoid and thyroid receptor; AAA: abdominal aortic aneurysm; eAAA: elective AAA; rAAA: ruptured AAA; lncRNA: long non-coding RNA; EC: endothelial cell; SMC: smooth muscle cell; ECM: extracellular matrix; SVB: Swiss Vascular Biobank; FFPE: formalin-fixed-in-paraffin-embedded; HE: Haematoxylin–Eosin staining; EvG: Elastica van Gieson staining; IHC: immunohistochemistry; RIN: RNA integration number (stage of RNA degradation); FGCZ: Functional Genomic Center Zurich; FDR: false discovery rate; CD45: cellular marker of T-cells; CD68: cellular marker of macrophages.
